# Finish the Good Work—A Plan Suggested to Regulate the Practice

**Published:** 1885-09

**Authors:** 


### FINISH THE GOOD WORK.

### *A Plan Suggested to Regulate the Practice.*

It will be observed that one of the avowed objects of this Journal is to lend its aid to completing the organization of the regular medical profession of Texas. Until some two years ago, in consequence of the rapidity with which the State is being filled, the influx of physicians, amongst others, into the State, as well as for the want of railroad communication, there was a sad lack of organization amongst medical men throughout Texas. Under the influence of Journalism, organization was stiumlated; many counties organizing into strong societies, sending delegates to swell the roll of the State Association, till now, Texas has a State Association second to few, in point of members, as well as material.

A good work has thus been begun, and it must be completed. We urge upon the members of the regular (Hippocratic) profession to organize! Wherever there are as many as half a dozen physicians, within a radius of ten miles, a nucleus of organization should be formed; and every legitimate effort made to bring into the pale of the influence of the State Association every reputable practitioner in that section. In no other way will it ever be possible to discriminate between the reputable and the disreputable — to distinguish the "sheep from the goats" as it were.

The profession should be convinced by this time that no aid can ever be expected from the Legislature towards "regulating the practice," unless a different method is adopted of asking it, and the regular profession of the State must itself regulate the practice. It can be done by inducing every physician, who has claims to respectability, to identify himself with a society, and thus with the State Society, which now, with an actual membership of five hundred, made up mostly of delegates from town and county societies on a basis of one delegate for every ten members, may be said to fairly represent the general profession of the State.

The day has come when public opinion demands that every practitioner should take his proper stand—show his hand. Those who are not for us, are against us. Thus, those who *cannot join* a County or State Society, would be branded, as it were; but the status of those who *can* do so but *will not*, is, and will ever be, equivocal—neither the profession nor the public knowing to which side they belong. Draw the line, and ask every one who is entitled to do so, to step over on the side of legitimate medicine.

The first step in the work of regulating the practice, is registration. We must have all reputable physicians registered, somehow and somewhere; and then the day may come when, not to be so registered—not to be a member of the organized branch of the profession—will be sufficient to mark a man as being unworthy of confidence, because inelegible to membership, or to registration. Thus will the profession have "become a law unto itself," as the good ministers say, and unto the people too; and thus will the practice be "regulated" in a very efficient manner. The line will be drawn between those who are, and those who are not, in affiliation with regular medicine ; the unworthy will be as effectually pointed out as if they had been rejected by an examining board, and *then* if people *will* employ them, they do so with their eyes open, and have no one to blame but themselves.

But that is not all ; nor is the above the only way in which the object of regulating the practice can be accomplished. When fully organized into town, county and district associations, and these are represented in the State Association, the profession can wield a powerful influence. It will then have an organized head, and its ramifications will extend in the remotest districts.

At a general convention, in addition to appointing a legislative committee to draft such a bill as is needed, let there be appointed a committee of two, three, or half a dozen, in every Senatorial district, whose duty it shall be to personally see and convince the representative of each county of the necessity of such legislation, to the end that such regresentative, when he goes to the legislative halls, will be thoroughly and correctly informed, not only of the numerous instances of malpractice by which lives are destroyed, but of all the facts bearing upon the subject of medical legislation ; and also informed that the profession of Texas, four thousand strong, organized, now speak as one man, and demand respectful attention. Thus, when the Legislature shall have assembled, the members will be prepared to vote intelligently, and not be subject to being influenced by every idle story, hatched up by interested persons ; nor carried away by spurious sympathy by those who claim to be persecuted for opinion's sake.

